# The multistep pathogenic hypothesis of amyotrophic lateral sclerosis is incompatible with the epidemiological data

**DOI:** 10.1007/s10654-025-01289-8

**Published:** 2026-01-24

**Authors:** Guglielmo Foffani, Daniele Urso, Josh Hiller, Marco Piccininni, Benoît Marin, Giancarlo Logroscino

**Affiliations:** 1 HM CINAC (Centro Integral de Neurociencias Abarca Campal), Hospital Universitario HM Puerta del Sur, HM Hospitales, Madrid, Spain; 2 Reina Sofia Foundation Alzheimer Center, CIEN Foundation, Instituto de Salud Carlos III, Madrid, Spain; 3 CIBERNED, Instituto de Salud Carlos III, Madrid, Spain; 4 Instituto de Investigación Sanitaria HM Hospitales, Madrid, Spain; 5 Center for Neurodegenerative Diseases and the Aging Brain, Department of Clinical Research in Neurology, University of Bari “Aldo Moro”, “Pia Fondazione Cardinale G. Panico”, Tricase, Lecce Italy; 6 Department of Mathematics and Computer Science, Adelphi University, Garden City, NY USA; 7 Institute of Public Health, Charité–Universitätsmedizin Berlin, Berlin, Germany; 8 Digital Health-Machine Learning Research Group, Hasso Plattner Institute for Digital Engineering, Potsdam, Germany; 9 Digital Engineering Faculty, University of Potsdam, Potsdam, Germany; 10 Sorbonne Université, INSERM, Institut Pierre Louis d’Epidémiologie et de Santé Publique, Équipe PEPITES, AP-HP, Hôpital Pitié-Salpêtrière, Département de Santé Publique, Centre de Référence sur les Agents Tératogènes (CRAT), Paris, France; 11 Department of Translational Biomedicine and Neurosciences (DiBraiN), University of Bari “Aldo Moro”, Bari, Italy

**Keywords:** Amyotrophic lateral sclerosis, Cancer, Epidemiology, Pathogenesis

## Abstract

Amyotrophic lateral sclerosis (ALS) is a multifactorial neurodegenerative disease whose incidence increases with age. According to the gene–time–environment hypothesis, ALS onset occurs through the interaction between genes and environmental exposures during ageing, which may involve a continuous accumulation process. Alternatively, the multistep pathogenic hypothesis, based on the Armitage-Doll multistep model from cancer research, posits that a discrete number of specific sequential “hits” are necessary to trigger ALS. Here we analyzed three large population-based epidemiological datasets of ALS to formally test whether the ALS age-incidence curve is better described by a power law, as predicted by the Armitage-Doll model, or by an exponential function, which is generally associated to continuous accumulation of damage and is incompatible with the Armitage-Doll model. We obtained moderate-to-extreme Bayesian evidence in favor of the exponential function compared to the power law. Cancer data were instead better aligned, as expected, with the power law. These results suggest that the multistep pathogenesis hypothesis based on the Armitage-Doll model cannot be extended from cancer to ALS, because it is incompatible with the epidemiological data. This calls for a re-consideration of the current understanding of ALS pathogenesis. Our work also warns against extending the Armitage-Doll multistep model from cancer to other aging-related diseases solely based on age-incidence curves.

## Introduction

Amyotrophic lateral sclerosis (ALS) is a neurodegenerative disease that affects both upper and lower motor neurons, leading to progressive muscle weakness, paralysis and ultimately death [1]. Despite extensive research, the multifactorial pathogenesis of ALS remains poorly understood. One intriguing characteristic of the disease (shared with other neurodegenerative diseases) is its age dependency, with the incidence increasing dramatically with age [2]. To explain this age dependency, the classic gene–time–environment hypothesis posits that ALS onset is driven by the interaction between genes and environmental exposures over the life course [3, 4]. This interaction may develop as an accumulation process that is more-or-less continuous. Alternatively, the more recent multistep pathogenic hypothesis suggests that a discrete number of specific sequential “hits” of genetic and environmental nature are necessary to trigger ALS onset [5]. The multistep pathogenic hypothesis arises from the observation that the relationship between ALS incidence and age appears to follow a “power law”, at least until relatively advanced ages, as evidenced by an approximately linear relationship between the two variables when they are both represented in logarithmic scale [5]. Adopting a model originally developed by Armitage and Doll in cancer epidemiology [6], this power law was suggested to reflect an underlying pathogenic process composed of discrete stochastic steps with specific characteristics [5]. The exact number of these steps supposedly depends on the presence of genetic mutations [7], geographical differences [8–10] and risk factors [11]. The multistep pathogenesis based on the Armitage-Doll model has gained increasing consensus for ALS [12, 13] and is being extended to other neurodegenerative diseases [14–17], and even to ageing-related diseases in which an underlying multistep process seems highly unlikely, such as Covid-19 [18]. However, the ALS multistep pathogenesis remains a hypothesis that still requires rigorous confirmation or falsification.

To draw an analogy familiar to clinicians, the inference problem addressed here is similar to the problem encountered in a differential diagnosis: observing certain signs that are compatible with a disease does not confirm the disease presence, as other diseases might produce similar signs. However, observing signs that are incompatible with the disease allows clinicians to exclude that disease from the differential diagnosis. Out of the analogy, the multistep pathogenic hypothesis of ALS was originally inferred from two main premises: (1) that an integer power law provides an accurate fitting of the age-specific incidence (i.e., the sign is present), and (2) that this accurate fitting is evidence, at least to some extent, for an underlying multistep pathogenesis according to the Armitage-Doll model (i.e., the sign is pathognomonic). Unfortunately, while it is true that the multistep pathogenic model described by Armitage and Doll would imply an integer power law relationship between incidence and age (i.e., the disease does produces the sign), the converse is not true (i.e., the sign is not pathognomonic). Indeed, different stochastic processes assuming different biological processes can lead to the same power law in the age-incidence curve [19, 20], so even a perfect fit of the power law does not provide conclusive evidence for a multistep pathogenesis (i.e., other disease produce the same sign). Conversely, if the age-incidence curve does *not* follow a power law, i.e. a falsification of the first premise, then the evidence would be *against* a multistep pathogenesis with the characteristics defined by Armitage and Doll (i.e. if the only sign present is not compatible with the disease, then the disease is not present). This reasoning allowed us to define a more stringent formal condition that is necessary – although not sufficient – for the validity of the multistep pathogenic hypothesis based on the Armitage-Doll model: not only the power law should fit the age-incidence curve [5, 7–11], but also the fitting performance of the power law should be superior against alternative functions of equivalent complexity (i.e. same number of degrees of freedom) that are incompatible with the Armitage-Doll multistep model.

**Fig. 1:**
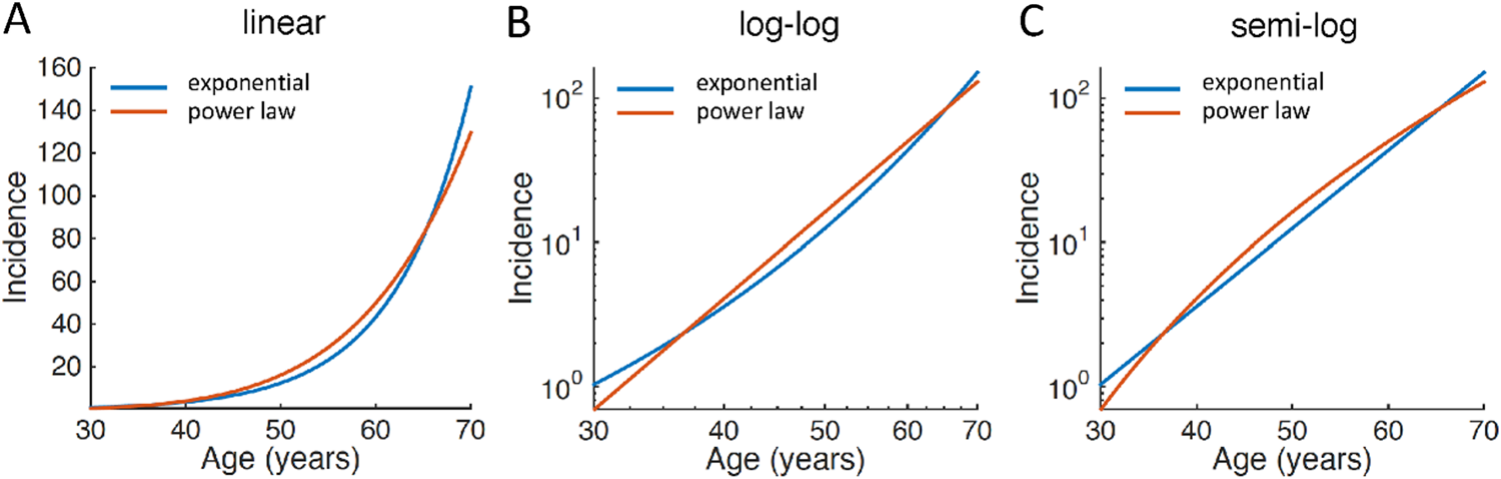
Theoretical exponential and power law curves of age-specific incidence. **A–C** Simulated exponential and power law curves, with arbitrary parameters, represented in **A** linear, **B** log-log and **C** semi-log scales. Exponential and power law curves cannot be easily differentiated in linear scale. However, the exponential curve is supralinear in log-log scale and linear in semi-log scale, whereas the power law curve is linear in log-log scale and sublinear in semi-log scale. As a reference, when noiseless data (in the 27–57 age range with 5-years blocks as in dataset 1) are truly exponential, linear fitting in log-log scale (i.e. assuming a power law) produces an R^2^ = 0.989, compared to R^2^ = 1.000 with a linear fitting in semi-log scale (i.e. assuming an exponential function), with an expected R^2^ difference of 0.011. Even with apparently high fitting performances, small R^2^ differences may be crucial to establish whether incidence increases with age as a power law, which is consistent with the Armitage-Doll multistep model, or as an exponential function, which is incompatible with the Armitage-Doll multistep model

In order to examine the validity of the hypothesis, here we analyzed three large epidemiological datasets of ALS from multiple registers, including the datasets originally employed to propose the multistep hypothesis [2, 5, 7–9]. We employed a Bayesian framework to formally test whether the age-specific incidence of ALS is better explained by a power law (linear in log-log scale) or by an exponential function (supralinear in log-log scale and linear in semi-log scale) (Fig. 1). The rationale for choosing as alternative the exponential function is that for at least two centuries it has being used to describe the age-specific mortality of the general population [21], it is generally associated to processes that consist of continuous accumulation of damage [22–25], and it is by definition incompatible with a multistep pathogenic process as defined by the Armitage-Doll model [6]. As a control analysis, we also applied the same approach to the cancer epidemiological data originally reported by Armitage and Doll in the paper that originated the multistep model [6].

## Methods

### Models

The multistep model from Armitage and Doll [6] describes a pathogenic process with the following characteristics [19]:

1. The pathogenic process is the result of several discrete changes with very low rates of occurrence;
2. Changes are stable (i.e. irreversible);
3. Changes must proceed in a unique order;
4. Changes occur independently (can occur in any order but only cause the disease if they occur in a specific order);
5. The probability of a specific change occurring in a given interval is only a function of the length of the interval and not when the interval occurs (i.e. independent increments).
6. The waiting time for the i-th step is exponentially distributed.
7. Almost surely, every person develops the disease (assuming an infinite lifespan).

If the pathogenic process has these characteristics, the relationship between incidence *I* and age *t* can be approximated (at least for a certain age range) as a power law

1 $$\:I\left(t\right)={a}_{m}{t}^{k-1}$$

where $\:{a}_{m}$ is a positive multiplicative constant that is proportional to the product of probabilities of each step and *k* is an integer that defines the number of steps (k > 1). Since

2 $$\:\mathrm{l}\mathrm{n}\left(I\right)=\mathrm{l}\mathrm{n}\left({a}_{m}\right)+\left(k-1\right)\mathrm{l}\mathrm{n}\left(t\right)$$

the power law (Fig. 1A) is linear in log-log scale with respect to age (Fig. 1B) and sublinear in semi-log scale (Fig. 1C).

A reasonable alternative to the power law for describing age-specific incidence is the exponential function, which is typically used to describe age-specific all-cause mortality [21], and it is generally associated to processes that consist of continuous accumulation of damage [22–25]. The exponential function describes the relation between incidence *I* and age *t* as

3 $$\:I\left(t\right)={a}_{e}{e}^{bt}$$

where $\:{a}_{e}$ is again a positive multiplicative constant and *b* is the degree of exponential acceleration (i.e. higher *b* implies faster increase with age). Since

4 $$\:\mathrm{l}\mathrm{n}\left(I\right)=\mathrm{l}\mathrm{n}\left({a}_{e}\right)+bt$$

the exponential function (Fig. 1A) is linear in semi-log scale (Fig. 1C) and supralinear in log-log scale (Fig. 1B). The exponential function is by definition incompatible with the multistep pathogenic process defined by Armitage and Doll [6]. Note that the exponential function implies a faster increase of incidence with age compared to the power law, and that the difference between the two functions is conceptually profound but visually subtle, so that a true exponential function would be fitted pretty well by a power law and viceversa. Consequently, R^2^ values are expected to be high for both functions when fitting real age-incidence data, and small R^2^ values differences become crucial to determine whether the age-incidence curve is truly a power law (thus compatible with the Armitage-Doll multistep model) or an exponential function (thus incompatible with the Armitage-Doll multistep model). Importantly, for both the power law (with k > 2) and the exponential function (with b > 0) the first and second derivatives are always positive, i.e. incidence accelerates with age, so the fitting performance of the two functions can be fairly compared only for age ranges in which the acceleration assumption is met. Violations of the acceleration assumption erroneously bias the fitting performance toward the power law. Moreover, in the Armitage-Doll multistep model, the power law mathematically arises as an approximation for low ages only. This means that, if the Armitage-Doll multistep model is true, the power law applies for age-incidence data in younger adults, but not necessarily in older persons [6, 26]. At high ages, where the acceleration assumption is not met and the Armitage-Doll multistep model’s age-incidence relationship becomes undefined, the data cannot be used to support the multistep model.

### Datasets

**Fig. 2:**
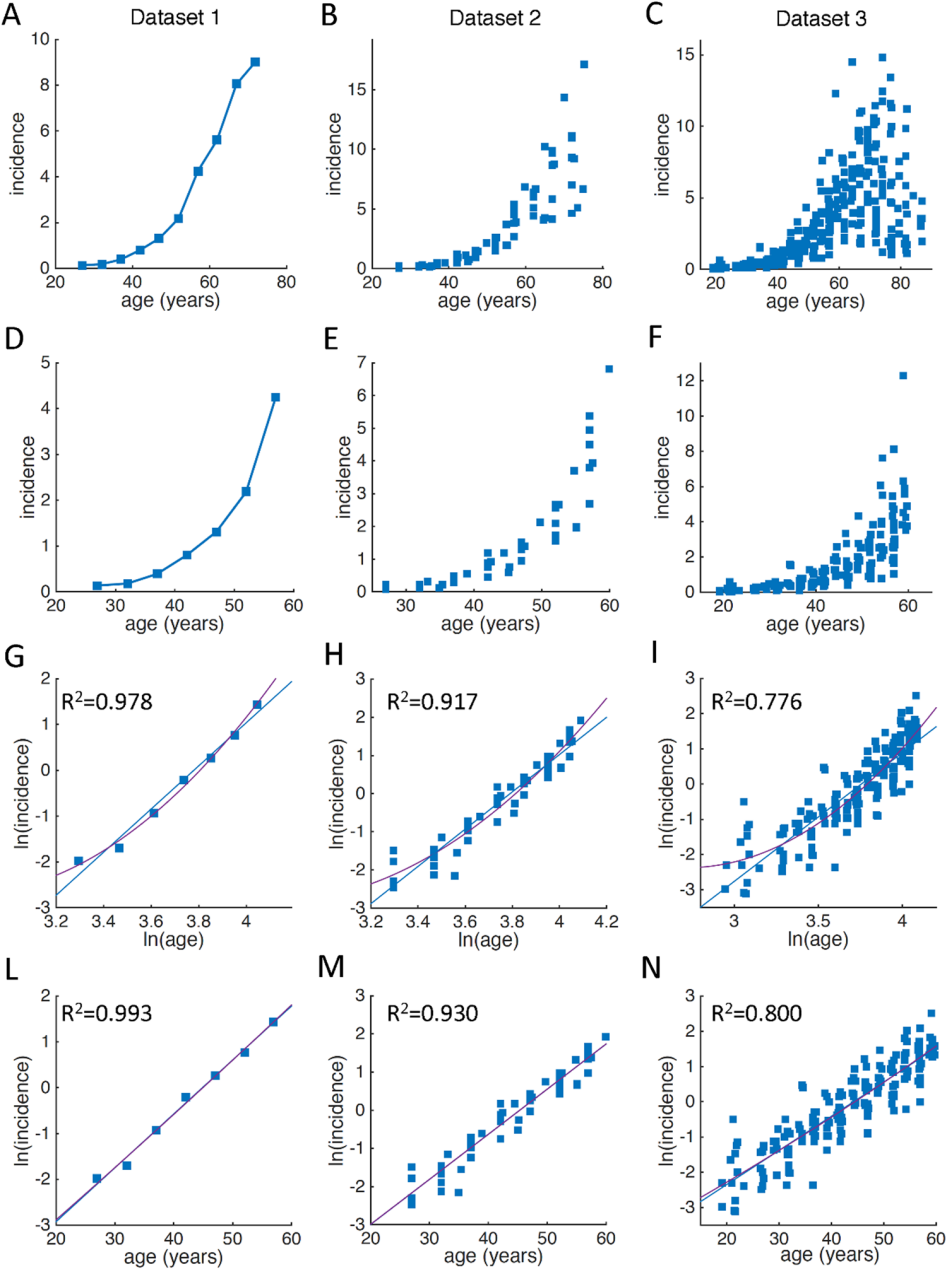
Comparison between power law and exponential function for ALS. **A–C** Age-specific incidence from three datasets, plotted with the full age range available. **D-F** Same data considering only the age ranges analyzed to meet the acceleration assumption. **G–I** Same data as in (**D**-**F**) represented in log-log scale, with linear fitting (blue) and quadratic fitting (purple). Note that the quadratic function provides a better fitting than the linear function in all datasets. (**L–N**) Same data as in (**D**-**F**) represented in semi-log scale, with linear fitting (blue) and quadratic fitting (purple). Note that the quadratic fit overlaps almost perfectly with linear fit, suggesting that the age-specific incidence of ALS is linear in semi-log scale. R^2^ corresponds to the linear fitting and is higher in semi-log scale in all three datasets. The increase of age-specific incidence of ALS is thus better described by an exponential function than a power law, which is not consistent with the Armitage-Doll multistep model

Three population-based datasets of age-specific incidence of ALS were analyzed in the present study:

1. Dataset 1 was the dataset of the original study proposing that ALS is a multistep process, which comprises a total of 6274 cases from 5 registers [5]. Age-specific incidence data were extracted by graphical reading of the figures (WebPlotDigitizer Version 4.6; https://automeris.io/WebPlotDigitizer/) individually for each register. For the main analyses, the data were pooled across registers in order to obtain an overall estimate for the 5 registers combined (Fig. 2A), as in the original work [5]. The graphical reading was validated by verifying that the extracted data were consistent with the results reported in the original paper (Table 1).
2. Dataset 2 included data from all studies suggesting that ALS is a multistep process published between November 2014 and April 2020 (total 19187 cases from 9 registers) [5, 7–9]. Age-specific incidence data were again extracted by graphical reading of the figures, but this time not pooled across registers due to the different age resolution (Fig. 2B). Again, the graphical reading was validated by verifying that the data were consistent with the results reported in the corresponding original papers (Table 2).
3. Dataset 3 included data from a large number of studies published until July 2016, systematically and meta-analytically reviewed in a previous paper [2]. For consistency with the previous datasets, age-specific incidence data were again extracted by graphical reading of the figures, without pooling across registers (Fig. 2C). The first and the last value of each register (i.e. incidence at the lowest and highest ages) were excluded because the corresponding age ranges were unbounded. Only adult age ranges (i.e. >18 years) and only non-zero incidences were included (because zero values, which can occur due to finite sampling, become minus infinity outliers in logarithmic scale). For dataset 3 we also had access to the original data, so the graphical reading was validated by verifying that the results obtained on the extracted data and the original data were equivalent.

As a control, we also analyzed the data of age-specific incidence of cancer originally reported by Armitage and Doll [6] (note that they additionally assumed that mortality was a good indicator for incidence). From the republished paper [27], we specifically extracted with graphical reading only the data for cancers that were consistent with a multistep pathogenesis, i.e. oesophagus, stomach, colon, rectum and pancreas. Again, the graphical reading was validated by verifying that the extracted data were consistent with the results reported in the original paper (Table 3), and we averaged the data across cancers for the main analyses.

**Table 1:** Validation of dataset 1

	Log-log slope estimate							
	All age groups included				First and last age groups omitted			
Register	Slope in the paper	Source	Slope after graph. reading	Source	Slope in the paper	Source	Slope after graph. reading	Source
England (SEALS)	4.7#	Table 2; Fig. 2	4.0	Sup. Figure 1	4.4	Sup. Table 1	4.4	Sup. Figure 1
Ireland	5.1	Table 2; Fig. 2	5.1	Sup. Figure 4	5.3	Sup. Table 1	5.3	Sup. Figure 4
Italy (Piedmont)	4.5	Table 2; Fig. 2	4.5	Sup. Figure 3	5.1	Sup. Table 1	5.1	Sup. Figure 3
Netherlands	4.6	Table 2; Fig. 2	4.6	Sup. Figure 2	4.9	Sup. Table 1	4.9	Sup. Figure 2
Scotland	5.0	Table 2; Fig. 2	5.0	Sup. Figure 5	5.8	Sup. Table 1	5.8	Sup. Figure 5
Overall	4.8	Table 2; Fig. 2	4.6##	Figure 1	5.1	Sup. Table 1	5.1	Figure 1

Age data extracted from the graphical reading were substituted with the exact age data provided in the original paper

#We detected an inconsistency between the total slope reported for England in Table 2/Fig. 2 (slope for Total and for Women cannot be identical) and the slope extracted from Suppl. Figure 1. We considered the latter (and the corresponding data) as the correct one

##We detected an inconsistency between the total slope reported for Overall in Table 2/Fig. 2 and the slope extracted from Fig. 1, which instead corresponded to the slope reported in Table 1 for Men. We thus considered Fig. 1 as not correct and reconstructed the overall total data by pooling the data from the 5 registers

**Table 2:** Validation of dataset 2

	Log-log slope estimate			
Register	Slope in the paper	Source	Slope after graph. reading	Source
Chiò et al. 2018 Genetic	4.7	Results, Fig. 1A	4.7	Figure 1A
Vucic et al. 2019 Australia	5.6	Results	5.6	Figure 2B
Vucic et al. 2020 Australia#	5.4	Figure 1A	5.4	Figure 1A
Vucic et al. 2020 Japan	5.4	Figure 1B	5.4	Figure 1B
Vucic et al. 2020 South Korea	4.6	Figure 1C	4.6	Figure 1C

Age data extracted from the graphical reading were substituted with the exact age data provided in the original papers, except for Vucic et al. 2019 and Vucic et al. 2020

#The Australia data in Vucic et al. 2019 and in Vucic et al. 2020 correspond to the same register. We used the data from Vucic et al. 2019 due to the greater age resolution

**Table 3:** Validation of armitage and doll cancer data

	Log-log slope estimate			
Register	Slope in the paper	Source	Slope after graph. reading	Source
Oesophagus (males)	6.3	Table 1	6.4	Figure 1
Stomach (males)	5.9	Table 1	5.9	Figure 1
Colon (males)	5.2	Table 1	5.2	Figure 2
Rectum (males)	5.6	Table 1	6.2#	Figure 2
Pancreas (males)	5.8	Table 1	5.7	Figure 1
Stomach (females)	5.3	Table 1	5.4	Figure 1
Colon (females)	5.0	Table 1	5.0	Figure 2
Rectum (females)	5.0	Table 1	5.0	Figure 2
Pancreas (females)	6.5	Table 1	6.5	Figure 1

#We detected an inconsistency between the slope reported for rectum cancer in males in Table 1 and the slope extracted from Fig. 2. We could not find an explanation for the inconsistency and considered the data shown in Fig. 2 as correct

### Main analyses

In all datasets, as main analyses we first linearly fitted the expected logarithm of the age-incidence data in log-log scale (Eq. 2, i.e. assuming a power law, as in previous studies proposing the multistep pathogenic hypothesis) and in semi-log scale (Eq. 4, i.e. assuming an exponential function), and calculated the corresponding fitting performances in terms of R^2^ values. We also performed a quadratic fitting in order to visually test whether the age-incidence data were supralinear in log-log scale (supporting the exponential function) or sublinear in semi-log scale (supporting the power law).

To more formally test whether the age-specific incidence data $\:{I}_{data}$ was linear in log-log scale (i.e. power law) or linear in semi-log scale (i.e. exponential), we performed the following log-linear multiple regression:

5 $$\:{\mathrm{ln}}\:\left( {I_{{data}} } \right) = \beta \:_{1} t\: + \beta \:_{2} {\mathrm{ln}}\left( t \right) + \beta \:_{3} + \varepsilon$$

where $\:\varepsilon \sim N(0,\sigma ^{2} )$ is a normally distributed error term. We thus estimated a Bayes factor (BF_10_) as posterior probability of the semi-log model (i.e. $\:{\beta\:}_{2}=0$, favoring exponential, H1) compared to the log-log model ($\:{\beta\:}_{1}=0,$ favoring power law, H0). This analysis was performed in JASP (version 0.14.0), which employs a Bayesian inference framework with non-informative or weakly informative priors, and utilizes Markov Chain Monte Carlo sampling for posterior estimation. We specifically used the default Jeffreys-Zellner-Siow (JZS) prior, which places a Cauchy distribution (scale = 0.354) for the standardized regression coefficients and an uninformative Jeffreys prior $\:p\left({\sigma\:}^{2}\right)\propto\:1/{\sigma\:}^{2}$ on the residual variance, and the beta binomial with a = 1 and b = 1 (i.e. uniform distribution on the number of predictors) for the model prior. In our setting, where only two mutually exclusive single-predictor models are compared, this ensures that the competing models have equal prior probability and thus posterior probability ratios and Bayes factors are equivalent.

### Ancillary analyses

To support the robustness of the main results, we performed the following ancillary analyses: Bayes factor robustness check, fractional polynomials, hierarchical mixed-effects models, leave-one-out cross-validation, sensitivity analyses, and validation on real data (dataset 3). The Bayes factor robustness check with JZS prior was performed in JASP. The remaining analyses were performed in Matlab, using the functions ‘fitlm’ for linear models and ‘fitlme’ for hierarchical mixed-effects models.

We first tested the robustness of the Bayes factors estimated in the log-linear multiple regression of the main analyses to the scale of the JZS prior, from very narrow (scale = 0.01) to very broad (scale = 100,000). We also estimated the Bayes factor BF_10_ as the maximum likelihood ratio between the semi-log model (i.e., exponential, H1) against the log-log model (i.e., power law, H0). While this maximum likelihood ratio does not represent a true Bayes factor (since it does not integrate over the prior), it can approximate it under certain conditions – namely, large sample sizes, regular and sharply peaked likelihoods, and flat (uniform) priors – where the marginal likelihood becomes dominated by the peak of the likelihood function. Note that the uniform prior can be seen as the limit of the JZS prior when the scale goes to infinity, so the maximum likelihood ratio can be considered as an additional robustness check with the widest prior compared to the JZS prior.

As a complementary approach, we also used fractional polynomials [28] to fit the age-incidence data with the Eq. 

6 $$\ln\!\left(I_{\mathrm{data}}\right)=\tilde{\beta}_{1}\, t^{(x)} \;+\; \tilde{\beta}_{2} \;+\; \varepsilon$$

with $\:{t}^{\left(x\right)}\equiv\:{t}^{x}$ when $\:x\ne\:0$ and $\:{t}^{\left(0\right)}\equiv\:\mathrm{l}\mathrm{n}\left(t\right)$, and *x* ranging from − 2 to 2 with 0.1 intervals. We estimated the optimal exponent *x*_opt_ that provided the best fitting of the data, as indicated by maximum likelihood. The expected optimal exponent is *x*_opt_=0 if incidence increases with age as a power law, whereas it is *x*_opt_=1 if incidence increases with age as an exponential function.

As an additional complementary approach, we implemented hierarchical models to account for the nested structure of the data, fitting mixed-effects linear models with random intercepts $\:{u}_{registry}$ and random slopes $\:{v}_{registry}$ for each register to capture the potential differences in the age-incidence relationship across registers. For the hierarchical power law model, we fitted the age-incidence data with the Eq. 

7 $$\:{\mathrm{ln}}\left( {{\mathrm{I}}_{{{\mathrm{data}}}} } \right) = \beta \:_{0} + \beta \:_{1} {\mathrm{ln}}\left( t \right) + (u_{{registry}} + v_{{registry}} {\mathrm{ln}}\left( {\mathrm{t}} \right)) + \,\varepsilon$$

For the hierarchical exponential model, we fitted the age-incidence data with the Eq. 

8 $$\:{\mathrm{ln}}\left( {{\mathrm{I}}_{{{\mathrm{data}}}} } \right) = \beta \:_{0} + \beta \:_{1} t + (u_{{registry}} + v_{{registry}} t) + \,\varepsilon$$

We estimated a Bayes factor BF_10_ as the likelihood ratio between the hierarchical exponential model (H1) against the hierarchical power law model (H0). Note that for this analysis the data were never pooled across registers.

In order to ensure robustness, we performed leave-one-out cross-validation on the linear log-log and semi-log regressions, as well as the hierarchical models, so that the contribution of each data point to the overall R^2^ value always comes from the prediction of a model constructed without that data point.

In order to assess to what extent the results depended on the exact age range considered in the analyses, we performed a sensitivity analysis comparing fitting performance (i.e. R^2^) between the exponential function and the power law when reducing age range (i.e. respecting the acceleration assumption) and when increasing the age range (i.e. violating the acceleration assumption, which is expected to bias the comparison toward the power law).

In dataset 3, all results obtained with the extracted data were corroborated on the real data.

Bayesian evidence in favor to alternative hypothesis (BF_10_ > 1) or to the null hypothesis (BF_01_ > 1) is described according to standard levels: anecdotal (1 < BF < 3), moderate (> 3), strong (> 10), very strong (> 30), and extreme (> 100).

## Results

### ALS age-incidence data cannot support the Armitage-Doll multistep model when incidence does not accelerate with age

We analyzed age-specific incidence data of ALS from three datasets (Fig. 2A-C). In all datasets, the incidence initially accelerated with age and then started decelerating, eventually even decreasing at the highest ages (dataset 3). Importantly, both the power law and the exponential function assume that incidence accelerates with age (i.e. positive second derivative). Consequently, the comparison between the two functions is valid and meaningful only for an age range in which this acceleration assumption is met. Note (i) that the acceleration assumption is in agreement with the original mathematical derivation of the Armitage-Doll multistep model, in which the power law was only proposed as an approximation of age-incidence data for younger adults and not for older persons [6, 26], and (ii) that violations of the assumption would bias the comparison toward the power law. In dataset 1 – where data could be pooled across registers, thereby reaching the smoothest age-incidence curve to estimate derivatives – the acceleration assumption was met for ages < 60 years. This is already an important result, because at higher ages the Armitage-Doll multistep model is undefined, so the data cannot support it. It thus remains to be determined whether the Armitage-Doll model holds for ages < 60 years. We thus used this age range – unless stated otherwise – to formally compare the multistep model against the exponential model in all three datasets (Fig. 2D-F).

### Main analyses 1: ALS age-incidence data is better fitted by an exponential function compared to a power law

When we fitted the data in log-log scale (i.e., assuming a power law), as in previous studies [5, 7–11], the performance of a linear regression was relatively high for the three datasets (R^2^_1_ = 0.979; R^2^_2_ = 0.918; R^2^_3_ = 0.776), which at first glance may seem to be consistent with the multistep pathogenic hypothesis based on the Armitage-Doll model. However, a quadratic fitting showed that the age-specific incidence was supralinear in log-log scale in all three datasets, which is not consistent with the power law (Fig. 2G-I). Accordingly, when we fitted the data in semi-log scale (i.e., assuming an exponential function), the performance of the linear regression was higher than in log-log scale for the three datasets (R^2^_1_ = 0.993; R^2^_2_ = 0.930; R^2^_3_ = 0.800) and the quadratic fitting was completely overlapping, suggesting that age-specific incidence was indeed almost perfectly linear in semi-log scale, and thus exponential (Fig. 2L-N). Note that the observed R^2^ differences (0.014, 0.012 and 0.024) may appear small, but are what one would expect when the true age-incidence curve is not a power law but an exponential function (Fig. 1).

### Main analyses 2: Bayesian evidence in favor of the exponential function compared to the power law in the ALS datasets

In order to more formally assess the evidence provided by the data in favor of the exponential function (H1) compared to the power law (H0), we implemented a Bayesian log-linear multiple regression (Eq. 5). This analysis confirmed that the age-specific incidence was not linear in log-log scale but in semi-log scale, favoring the exponential function against the power law with moderate-to-extreme evidence (BF_10_1_ = 8.7; BF_10_2_ = 43.5; BF_10_3_ = 2.3⨯10^4^). The estimated rate of exponential increase (reported with corresponding 95% credibility interval) was similar in the three datasets: b_1_ = 0.117 (0.106–0.128), b_2_ = 0.118 (0.108–0.127), b_3_ = 0.114 (0.098–0.130). These results suggest that an exponential function provides a better description of the ALS age-incidence data compared to a power law, which is *not* consistent with the Armitage-Doll multistep model.

### Ancillary analyses on ALS datasets

The exact level Bayesian of evidence is dependent on the choice of the prior. In the Bayesian log-linear multiple regression above we employed the default JSZ prior, which assumes that the regression coefficients are Cauchy distributed with scale 0.354. We thus verified that the Bayes factors supporting the exponential function against the power law were robust to the scale of the JSZ prior, from extremely narrow (scale 0.01: BF_10_1_ = 8.0; BF_10_2_ = 43.5; BF_10_3_ = 2.3⨯10^4^) to extremely wide (scale 100,000: BF_10_1_ = 27.0; BF_10_2_ = 50.0; BF_10_3_ = 2.7⨯10^4^). In the limit of the scale approaching infinity, the JZS prior tends to the uniform prior, bringing the estimated Bayes factor closer to the approximation as maximum likelihood ratio from the simple linear regressions in log-log and semi-log scales (BF_10_1_ = 46.2; BF_10_2_ = 58.9; BF_10_3_ = 2.9⨯10^4^). Note that the maximum likelihood ratio approximation tends to overestimate the level of evidence compared to the default JSZ prior. This is more noticeable with small numbers of points (e.g., in dataset 1 on pooled data, where the level evidence increased from moderate to very-strong) and becomes less noticeable or negligible with larger numbers of points (e.g., in dataset 2 and dataset 3, where the level of evidence remained essentially unaltered in the very-strong or extreme range, respectively). Acknowledging the overestimation, approximating Bayes factors as maximum likelihood ratios is computationally convenient and was thus adopted in the more complex ancillary analyses below. Still, independently on the exact choices for estimating Bayes factors, all results favored the exponential function compared to the power law.

As a complementary approach to estimate the exact function describing the age-incidence curves, we used fractional polynomials (Eq. 6). This approach does not perform a comparison between two competing functions, but instead optimizes the fitting performance of a polynomial equation with a fractional exponent *x* that is allowed to vary across a relatively large range of values, including as special cases the exponential function (x = 1) and the power law (x = 0). We found that the optimal exponent for fitting the age-incidence data was remarkably consistent with an exponential function (*x*_opt_1_ = 1.1; *x*_opt_2_ = 1.0, *x*_opt_3_=1.2).

In order to account for the variability between registers, we also analyzed the data with hierarchical mixed-effects models (Eq. 7 and Eq. 8). The fitting performances of the power law (R^2^_1_ = 0.942; R^2^_2_ = 0.958; R^2^_3_ = 0.895) were again inferior to the exponential function (R^2^_1_ = 0.957; R^2^_2_ = 0.969; R^2^_3_ = 0.914). The Bayes factors estimated from the maximum likelihood ratios again supported the exponential function against the power law with very-strong-to-extreme evidence (BF_10_1_ = 31.8; BF_10_2_ = 228.2; BF_10_3_ = 8.1⨯10^5^). Note that in dataset 1 the linear regressions were performed on pooled data whereas the hierarchical models were fitted on non-pooled data.

With leave-one-out cross-validation, the fitting performances of the simple linear regressions in log-log and semi-log scales were somewhat lower, as expected, but they confirmed that the fitting performances of the power law (R^2^_1_ = 0.932; R^2^_2_ = 0.908; R^2^_3_ = 0.770) were inferior compared to the exponential function (R^2^_1_ = 0.984; R^2^_2_ = 0.923; R^2^_3_ = 0.795). Similar findings were obtained with leave-one-out cross validation of the hierarchical mixed-effects models, again showing that the fitting performances with the power law (R^2^_1_ = 0.904; R^2^_2_ = 0.916; R^2^_3_ = 0.811) were inferior compared to the exponential function (R^2^_1_ = 0.930; R^2^_2_ = 0.941; R^2^_3_ = 0.851).

**Fig. 3:**
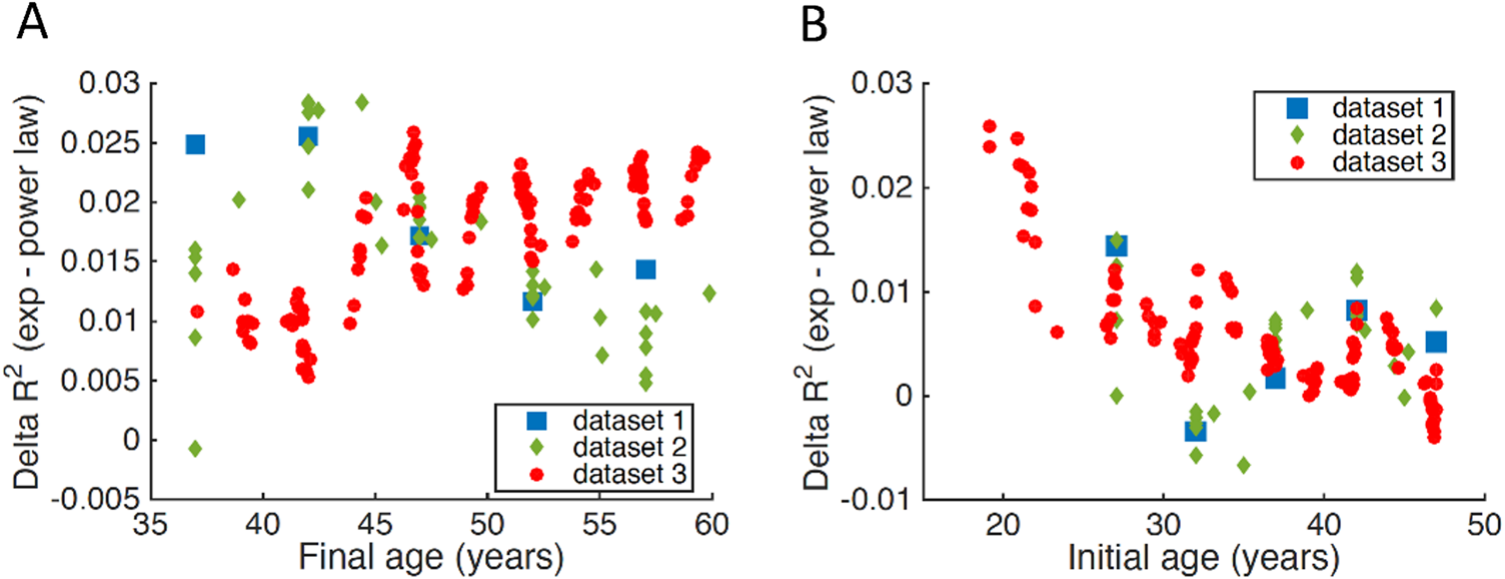
Sensitivity analysis. **A**, **B** Difference in fitting performance (i.e. R^2^) between the exponential function and the power law when either decreasing the final age (**A**) or increasing the initial age (**B**). Positive values indicate better performance of the exponential function

Within the age range that met the acceleration assumption (i.e. <60 years), the sensitivity analysis suggested that the exponential function was generally superior to the power law independently of the exact final age (Fig. 3A) or initial age (Fig. 3B) included in the analyses. The superiority of the exponential function progressively decreased as the initial age included in the analyses increased (Fig. 3B), possibly reflecting a decrease of the acceleration of incidence with age, anticipating the deceleration at older ages, and/or the decrease of data points available. Even with violation of the acceleration assumption including ages ≥ 60 years, which is expected to progressively bias the comparison in favor of the power law, the fitting performance of the exponential function remained superior to the power law until 67, 65 and 72 years for datasets 1, 2 and 3, respectively.

Finally, in dataset 3 we obtained results consistent with the extracted data when the analyses were performed on the original data, with extreme evidence favoring the exponential function compared to the power law both for the log-linear multiple regression (BF_10_3_ = 1.3*10^4^), the simple linear regressions in log-log and semi-log scale (BF_10_3_ = 1.5⨯10^4^), the fractional polynomials (*x*_opt_3_ = 1.2) and the hierarchical mixed-effects models (BF_10_3_ = 1.2⨯10^5^).

### Control analyses: Bayesian evidence in favor of the power law compared to the exponential function in the cancer dataset

**Fig. 4:**
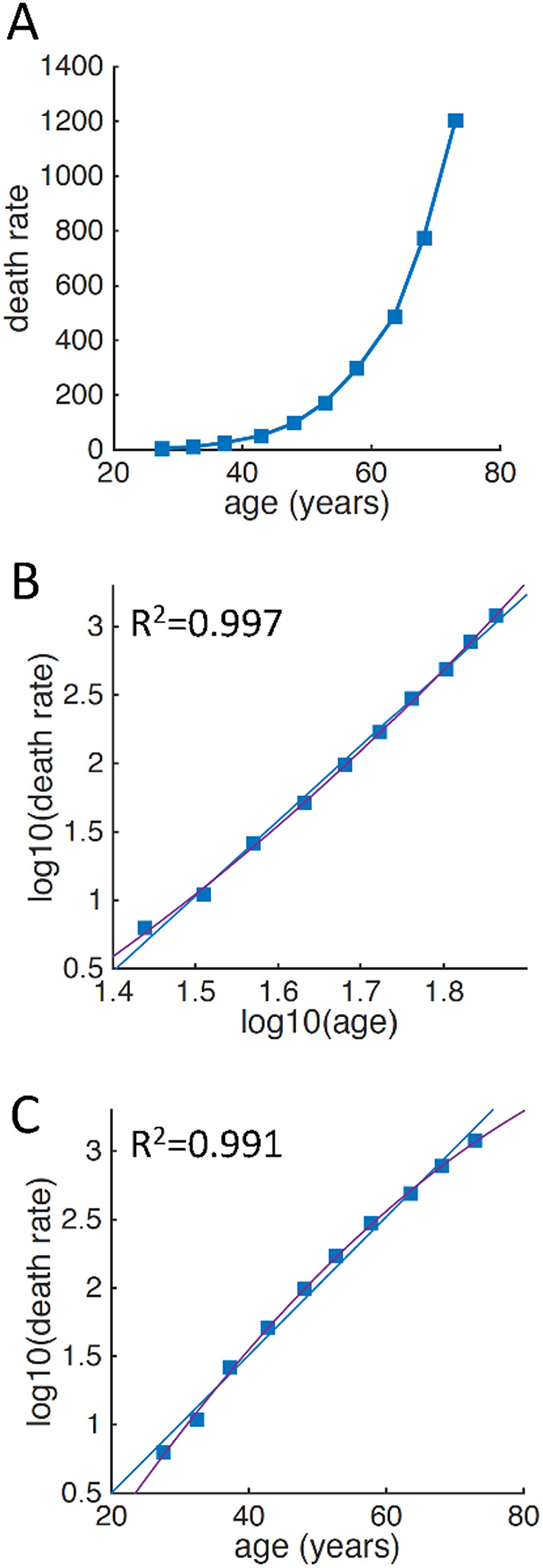
Comparison between power law and exponential function for cancer. **A** Age-specific average death rate. **B** Same data as in (**A**) represented in log-log scale, with linear fitting (blue) and quadratic fitting (purple). **C** Same data as in (**A**) represented in semi-log scale, with linear fitting (blue) and quadratic fitting (purple). The base-10 logarithm is used for here consistency with the original paper. Note that the quadratic function clearly provides a better fitting than the linear function in semi-log scale. R^2^ corresponds to the linear fitting and, differently from ALS, is higher in log-log scale. The increase of age-specific mortality (used by Armitage and Doll as a proxy for incidence) of cancer is thus better described by a power law than by an exponential function, which is consistent with the Armitage-Doll multistep model

For the pooled cancer data, the acceleration assumption was met for the full range of available ages (Fig. 4A). The age-specific mortality was indeed more linear in log-log scale (Fig. 4B) than in semi-log scale (Fig. 4C), expectedly favoring the power law against the exponential function with strong Bayesian evidence (BF_01_ = 25.8). The result was robust to the scale of the JSZ prior, from extremely narrow (scale 0.01: BF_01_ = 25.8), to extremely wide (scale 100,000: BF_01_ = 66.7), to the asymptotic limit of the uniform prior (BF_01_ = 105.3). The optimal exponent for fitting the data with fractional polynomials (*x*_opt_ = 0.4) was more consistent with a power low (x = 0) compared to an exponential function (x = 1). The optimal exponent became even closer to the theoretical power law (*x*_opt_ = 0.3) when the fractional polynomial fitting was performed with hierarchical mixed-effects models on non-pooled data (BF_01_ = 7.6⨯10^10^). Note that a Bayes factor several orders of magnitudes larger in hierarchical models using non-pooled data compared to the regressions on pooled data is expected when the main source of variability lies between rather than within registers (which was the case for the cancer data but not in the ALS dataset 1).

## Discussion

Our results show that, differently from cancer, the increase of ALS incidence with age is better described by an exponential function compared to a power law. These findings question the plausibility of all pathogenic processes of ALS inducing a power law for the age-incidence relationship. The multistep pathogenic hypothesis of ALS based on the Armitage-Doll model is thus not supported by the available epidemiological data.

The multistep model was introduced in cancer epidemiology with the seminal paper by Armitage and Doll in 1954 [6]. The identification of the causal mechanisms underlying the pathogenic process of a disease is a task that heavily involves the integration of prior knowledge by triangulating different types of evidence, and it cannot be reduced to a curve fitting problem, as clearly shown by the debate around carcinogenesis in the 1950 s [6, 29, 30], a debate that is still quite active in the new millennium [31–34]. In the words of Armitage himself: “In the construction of mathematical models for biological phenomena it is not uncommon to find that theories of quite disparate types provide good fits to the same data. Discrimination between models must then depend partly on general biological plausibility and partly on the ability of the models to explain new data” [26]. Indeed, in their original paper, Armitage and Doll clearly state that several other diseases (such as cerebral haemorrhage, coronary thrombosis and gastric ulcer) follow an age-incidence relationship similar to the power law, but they would consider unreasonable to believe that the same multistep biological mechanism is at play for these diseases [6]. In retrospect, an important reason why their model has formed the basis for most, if not all, modern models of carcinogenesis is because it was able to explain many sources of laboratory and epidemiological data, not just incidence. In particular, it explained why in experiments with mice cancer was able to be induced when a “promoter” carcinogen was applied after an “initiator” carcinogen but not the other way around; their model predicted dose response for people who were often exposed to low dose carcinogens; and their model explained why people would develop cancer much later than the time they were exposed to carcinogens. Their model provided a coherent explanation for decades of laboratory and epidemiologically significant evidence. Our new analyses on the Armitage and Doll epidemiological data indeed confirm that the increase of cancer incidence with age is best described by a power law compared to an exponential function, which is consistent with the original multistep model of carcinogenesis.

**Fig. 5:**
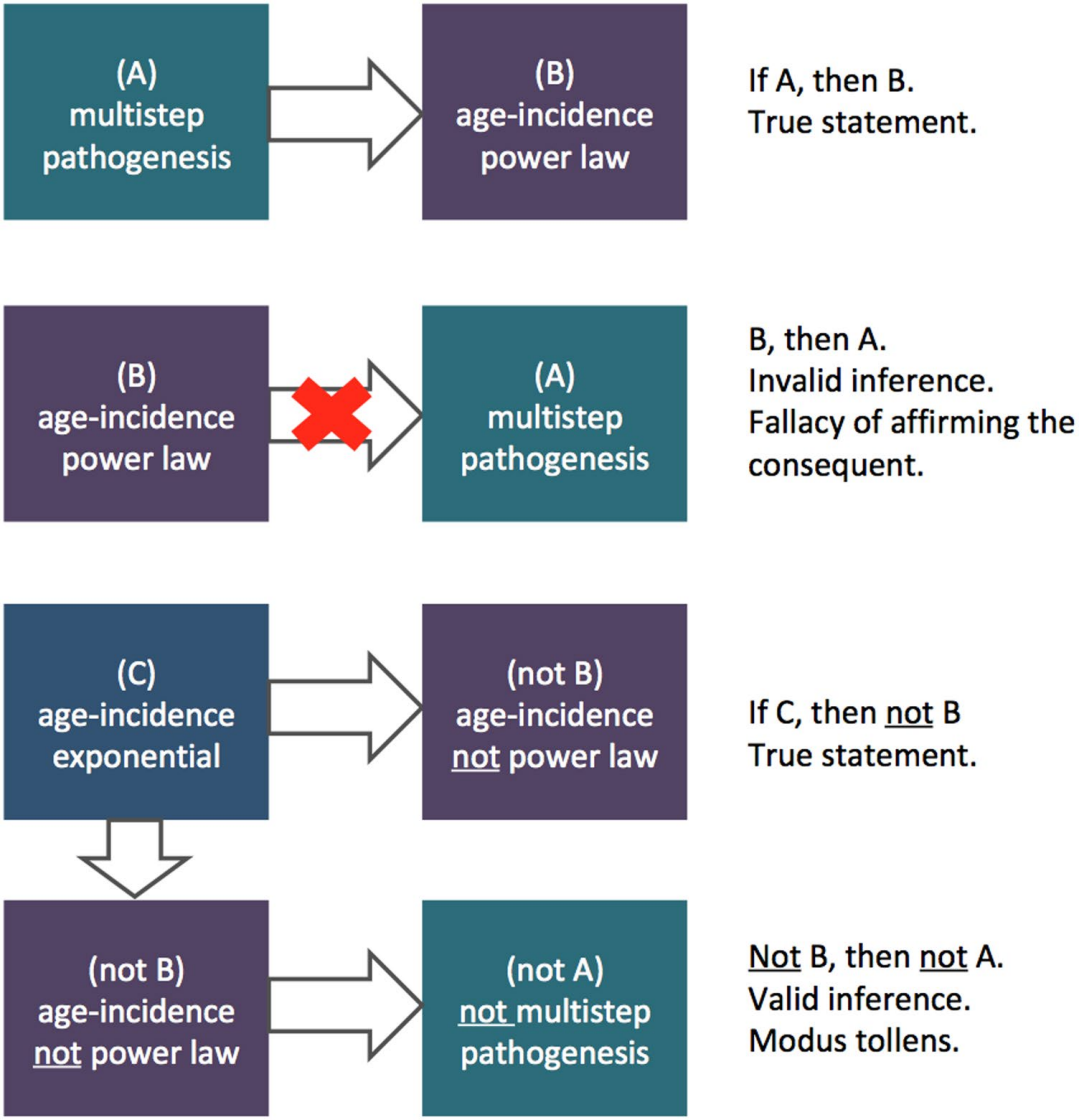
Logical inferences leading to the falsification of the multistep pathogenic hypothesis of ALS. In the figure, multistep pathogenesis specifically refers to the pathogenesis defined by the Armitage-Doll multistep model

The multistep pathogenic hypothesis was extended from cancer to ALS with an influential paper published by Al-Chalabi et al. in 2014 [5]. The hypothesis has now been referenced in several important reviews and studies, underscoring its significance in shaping the discourse around ALS etiology and progression [12, 13]. In the original paper, Al-Chalabi et al. acknowledged that their findings “do not definitively prove ALS as a multistep process” [5], but subsequent works have been more assertive in their support for the hypothesis, explicitly stating that “ALS is a multistep process” [8, 9]. This ontological statement corresponds to the common fallacy of affirming the consequent: if ALS is an Armitage-Doll multistep process, then the age-incidence curve should be well fitted by the power law but, unfortunately, the converse direction of causality does not hold (e.g. if it rains, the grass will be wet; but if the grass is wet, it doesn’t necessarily mean that it has rained) (Fig. 5). Our new analyses on the available epidemiological data show that the increase of ALS incidence with age is *not* best described by a power law, but by an exponential function, which is thus not consistent with the multistep hypothesis of ALS as defined by the Armitage-Doll model.

Our findings have methodological, biological and translational implications. First, since infinite functions can pass through a finite set of data points, and a function is generally compatible with several biological processes, trying to infer the pathogenic process from a curve-fitting exercise is a task doomed to fail. Researchers should instead formulate pathogenic models based on biological plausibility, derive mathematically the induced age-incidence relationship, and only then, compare the expected behavior in the age-incidence relationship with the real epidemiological data. This framework allows for rejecting false biological models, and leads to advancement in research. Second, even though proving that a mathematical function fits the age-incidence curve does not prove the intended biological model (i.e. if the grass is wet, we cannot conclude it has rained), showing that a different function with equivalent complexity actually fits better does challenge the biological model, at least in its specific formulation (i.e. if the grass is not wet, it didn’t rain) (Fig. 5). The comparison between functions is particularly informative when a benchmark is used. In our application, we used as a benchmark the exponential function, which is typically employed to describe processes, such as all-cause mortality, that are not believed to be a sequence of discrete steps but rather a continuous accumulation of damage with ageing [22–25]. The worse fit of the power law compared to the exponential function suggests that ALS is *not* a multistep pathogenic process as described by the Armitage-Doll model (Fig. 5). And more generally our results suggest that ALS pathogenesis is not compatible with any biological process inducing a power law in the age-incidence curve. Third, our work provides a strong note of caution against extending the multistep pathogenic hypothesis from cancer to any other diseases solely based on age-incidence curves. For example, a similar superior performance of the exponential function compared to the power law was obtained when fitting the age-incidence curve of Parkinson’s disease [35]. Paradigmatic examples of conceptual misapplications are Covid-19 [18], where death rate is admittedly exponential, or Huntington’s disease, where the CAG expansion in the HTT gene is the definitive pathogenic cause of the disorder, but because the mutation is inherited and present from birth, it does not constitute a stochastic step in the sense defined by the Armitage-Doll framework. More generally, it is important to emphasize that inherited genetic mutations do not properly qualify as “steps” in the Armitage-Doll model, which is predicated on the sequential occurrence of rare, stochastic changes acquired during life. Fourth, until a valid alternative becomes available, translational efforts in ALS should rely on the gene–time–environment hypothesis [3, 4], interpreted as a continuous process of damage accumulation, for which biological evidence exists. The incidence of ALS displays a degree of geographical heterogeneity that cannot be fully explained by the known risk factors [2, 36], prompting for the identification of modifiable risk factors in the exposome and the investigation of gene-environment interactions [13]. More research, and possibly more models, will be necessary to achieve a full biological integration of genetic and environmental factors for the development of preventive and therapeutic treatments of ALS.

Few final considerations should be devoted to the main limitations of the present study. First, all the main analyses are based on data extracted with graphical reading. Yet, we validated the graphical reading with the results of the original papers (datasets 1, 2 and cancer data) and with the original data (dataset 3). Second, dataset 3 is admittedly more noisy, as it includes registers with lower quality. Nevertheless, this should reduce the fitting performance within models, but is unlikely to change the direction of the evidence in the comparison between models. Third, in the main analyses we did not explicitly consider the variability of age-specific incidence between registers. However, all results were confirmed in the ancillary analyses in which the variability was explicitly considered with hierarchical mixed-effects models. Fourth, we did not perform any separate analyses accounting for sex-related differences or geographical differences in ALS incidence. These differences will deserve detailed further investigations. Fifth, our results are valid only for the age range in which incidence accelerates with age, which is assumed by both the exponential function and the power law. At higher ages, the Armitage-Doll multistep model is undefined, so the data cannot support it. The conclusion that the multistep pathogenic hypothesis of ALS based on the Armitage-Doll model is not supported by the epidemiological data is thus valid for all ages. To describe the deceleration, peak and later decrease of ALS incidence at higher ages, the exponential function could be extended to more complex functions already explored in the cancer literature and for Parkinson’s disease, such as the beta model or the susceptibility model [16, 25, 35, 37].

In conclusion, our results suggest that the multistep pathogenic hypothesis based on the Armitage-Doll model developed in cancer research cannot be extended to ALS. The ALS epidemiological data are better described by an exponential function compared to a power law, which is incompatible with the Armitage-Doll multistep model. This conclusion underlines the importance of focusing on pathogenic mechanisms of ALS, in order to efficiently direct research efforts toward the full biological understanding of genetic and environmental interactions for the development of preventive and therapeutic treatments of ALS.
